# A national survey of knowledge, attitude, practice, and barriers towards pharmacovigilance and adverse drug reaction reporting among hospital pharmacy practitioners in the United Arab Emirates

**DOI:** 10.1186/s40545-023-00593-6

**Published:** 2023-07-18

**Authors:** Sawsan Shanableh, Hadzliana Zainal, Muaed Alomar, Subish Palaian

**Affiliations:** 1 Discipline of Clinical Pharmacy, School of Pharmaceutical Sciences, Universiti Sains Malaysia, Pulau Pinang, Malaysia; 2 Department of Clinical Sciences, College of Pharmacy and Health Sciences, Ajman University, Ajman, United Arab Emirates

**Keywords:** Pharmacovigilance, ADR reporting, Hospital pharmacists, Knowledge, Attitude, Practice, Barriers, United Arab Emirates

## Abstract

**Background:**

Pharmacovigilance (PV) is an essential component of patient safety and pharmacists are expected to be aware of the PV processes and willing to report ADRs. This study assessed the hospital pharmacists’ knowledge, attitude, and practice toward PV, barriers faced by them in ADR reporting, and factors influencing ADR reporting.

**Method:**

A cross-sectional nationwide questionnaire survey was conducted among randomly chosen hospital pharmacists across UAE from March to July 2022. The filled questionnaires were assessed both descriptively [median (IQR scores), maximum 5 for Likert type and 1 for knowledge questions] and inferentially using the Mann–Whitney *U* test (for dichotomous variables) and the Kruskal–Wallis test (for variables with more than two responses) at alpha value = 0.05. Post hoc analyses and correlations were performed wherever applicable.

**Results:**

Of the 342 respondents, the majority were knowledgeable about the concepts of PV (93.3%; *n* = 319) and ADRs (86.8%; *n* = 297). The overall median (IQR) knowledge score was 5 (3–7)/9. Knowledge levels within ‘qualification groups’ varied significantly (*p*-value < 0.001) and participants ‘between 10 and 14 years of experience’ had more knowledge than those ‘with < 5 years of experience’ (*p*-value < 0.001, Bonferroni test). The overall median (IQR) attitude score was 22 (20–24)/30. Most respondents (90.6%; *n* = 311) were willing to spare time to review patients’ ADR reports. The overall median (IQR) practice score was 17.5 (11–21)/24. Although 71.1% (*n* = 243) noticed ADRs during the previous year, only 53.2% (*n* = 182) reported an ADR, the reasons for underreporting being mainly due to a lack of proper training [median IQR score 4(4–5)/5]. The ‘clinical pharmacists’ engaged themselves more in pharmacovigilance than ‘pharmacists’ (*p*-value =  < 0.001), and ‘inpatient pharmacists’ reported more ADRs than ‘pharmacists’ (*p*-value = 0.018); Bonferroni test. The overall median (IQR) barrier score was 26 (23–29)/40 and the common barrier was ‘lack of awareness about the national ADR reporting system 4 (4–5)’. The pharmacists in this study suggested incentives for reporting ADRs (69.3%; *n* = 237).

**Conclusion:**

The authors concluded professional training courses for practicing pharmacists and educational curriculums related to PV and ADR reporting processes are to be considered for future pharmacists in order to inculcate ADR reporting culture and practices.

**Supplementary Information:**

The online version contains supplementary material available at 10.1186/s40545-023-00593-6.

## Introduction

Adverse drug reactions (ADRs) contribute significantly to increasing the levels of morbidity and mortality globally [1]. The World Health Organization (WHO) defined ADRs as “a response which is noxious and unintended, and which occurs at doses normally used in humans for the prophylaxis, diagnosis, or therapy of disease, or for the modification of physiological function” [2]. Several studies reported the consequences of ADRs which varied from socio-economic to health-related problems [3–5] and it can be ended by death [6]. It is one of the major causes of hospitalization, accounting for (5–13%) of cases and increasing to 24% in elderly patients [7–9].

Reporting ADRs is one of the main pharmacovigilance (PV) methods that aim to assure medications' safety and protection from harmful events [1]. It is critical for the early detection of serious and uncommon ADRs, as well as for guiding causality hypotheses, investigation priorities, and regulatory measures [10]. However, it depends on the adequate knowledge, attitude, and practices of the healthcare providers toward ADR reporting. Previous studies conducted in different countries in this field reveal that there was a deficiency in the knowledge level about PV among pharmacists and more awareness about PV and ADRs reporting were needed [1, 11, 12]. On the other hand, other studies showed that pharmacists have enough knowledge about PV and ADRs reporting [6, 10, 13–17]. More studies found that, despite high levels of knowledge about PV and ADRs reporting, there was still underreporting, which was due to pharmacists' unfamiliarity with the reporting process or due to being unaware of the national ADR monitoring systems [6, 9, 12, 13, 18–20]. A survey from eight Middle East countries (e.g., Saudi Arabia, Qatar, Bahrain, Oman, Yemen, Jordan, Egypt, and Lebanon) reported PV programs in those countries are not well developed and have only a limited regional collaboration [21]. Under-reporting of ADRs delays early recognition of ADRs and thereby increasing the morbidity and mortality. Identifying the factors influencing reporting is critical for proposing measures to improve the system. Pharmacists play an important role in the detection, assessment, and spontaneous reporting of ADRs [22]. As a result, educating pharmacists to improve their PV knowledge, attitude, and practice is critical in developing strategies to encourage ADR reporting [1].

Healthcare in UAE is regulated by the Ministry of Health (MOH), the Department of Health-Abu Dhabi (DOH), and the Dubai Health Authority (DHA)-Dubai. These regulatory bodies are constantly working to raise awareness among healthcare professionals about the importance of monitoring and reporting ADR in the country [9]. Several policies and legislations have been drawn up regarding this issue; nevertheless, the proper application remains of concern [17].

A comprehensive literature review shows no focused studies in the UAE on the role of hospital pharmacists in ADR reporting had been done. Hence, this study was conducted to assess the hospital pharmacists’ knowledge, attitude, and practice toward PV, barriers faced by them in ADR reporting, and factors influencing ADR reporting.

## Methodology

### Study design

A cross-sectional quantitative study was conducted among hospital pharmacy practitioners in several hospitals across the country. The questionnaire was distributed electronically via Google Survey. The study design and write-up were built using the CHERRIES Checklist [23]. Each participant is only permitted to respond to the survey once because it requires an email sign-in before it can be started. The study was conducted over five months; from March to July 2022.

### Ethical considerations

The study had been approved by Ajman University Research Ethics Committee (Approval Reference number: P-H-F-7-Jan), Dubai Scientific Research Ethics Committee (Approval Reference number: DSREC-SR-03/2022_02) and Universiti Sains Malaysia Research Ethics Committee (Approval Reference number: USM/JEPeM/22070495). Participants were told that by responding to this survey, they were agreeing to participate in the study, but they could stop at any time. The consent form was included in the survey. The codes were utilized in the study without mentioning any names, and all data acquired were handled in a highly private manner. The researchers complied with all the rules established by the ethical review board.

### Study participants and sample size

Pharmacists from different hospitals were invited to take part in this study. All 7 emirates were included in the study representing the whole nation. Cochran’s Formula was used to calculate the sample size based on the number of licensed hospital pharmacists in the country. The target population was calculated using published data showing the total number of pharmacists in Dubai Health Authority (DHA) from 2010 to 2019 [24] and the population in each emirate because the total number of pharmacists working in hospital pharmacies in the UAE remained unknown. In order to determine the licensed hospital pharmacist in each emirate in relation to the population in each emirate, the percentage of licensed hospital pharmacists in Dubai in proportion to the total population in the same emirate was calculated. Consequently, there are approximately 12,257 licensed pharmacists in the UAE. Using the above-mentioned equation and considering that the margin of error is 5% (CI = 95%), the response distribution is 50% and the calculated sample size was 386.

### Sampling procedure

In this research in order to achieve a nationwide proportional representation, a non-probability quota sampling method was followed. The estimated sample was divided among the number of pharmacists working in hospitals in each of the seven emirates. Conveniently, pharmacists working in hospital pharmacies were chosen in accordance with their enthusiasm in participating and professional networks. Table 1 displays the predicted sample size.

**Table 1:** Sample distribution among the seven emirates (*n* = 386)

Name of the emirate	No. of licensed pharmacists required
Dubai	145
Abu Dhabi	122
Sharjah	75
Ajman	17
Ras Al Khaimah	14
Fujairah	11
Umm Al Quwain	2

### Inclusion and exclusion criteria

The study was opened to any licensed pharmacists employed by any hospital within the seven emirates. The study did not include any additional pharmacists who worked in settings other than hospitals.

### Study tool

#### Development of the structured questionnaire

The questionnaire for this study was developed after reviewing other studies on the same subject extensively [1, 10, 13–16, 25–28] and after discussion among the co-authors.

The study tool had six sections which included sociodemographic data, knowledge about pharmacovigilance and adverse drug reaction reporting, attitude and practice towards ADR, barriers that may affect implementing PV and the last section about suggestions that may enhance ADR reporting.

Participants’ KAP levels were defined as ‘low’, ‘moderate’, and ‘high’ based on Bloom's cutoff point [29]. The sociodemographic section (Sect. 1) had nine questions with short answer responses. Section 2 had nine multiple-choice questions which is testing the knowledge level of pharmacists about PV and ADR reporting. Each question was scored with one point for the correct answer and zero for the wrong answer. Responses with scores < 3 points were categorized as ‘low’, 3–5 points ‘moderate’, and > 5 points ‘high’. The attitude (Sect. 3) was assessed by 6 Likert scale questions to measure the level of agreement and each answer had five options ‘strongly disagree, disagree, neither agree nor disagree, agree and strongly agree’. The scores ranged from 6 to 30 points. Results with scores < 8 points were grouped ‘low’, 8–15 points ‘moderate’, and > 15 points ‘high’. Section 4 (practice) was ‘Yes, No’ questions with a total of 8 statements. Section 5 is about the barriers faced the hospital pharmacists in ADR reporting. It contained 8 Likert scale statements with 5 responses starting with ‘strongly disagree’ and ending with ‘strongly agree’. The result of scores ranged from 8 to 40. The last section, Sect.  6 assessed the factors that would encourage the pharmacists to report an adverse drug reaction. This section contained 11 Likert scale statements with 5 responses starting with ‘strongly disagree’ and ending with ‘strongly agree’. The result of scores ranged from 11 to 55.

#### Content validity

Content validation had been done by sending the questionnaire with content validation form to three experts asking them to express their opinions and writing down their comments related to each question. These experts include professors employed in UK and Malaysian universities, an associate professor at a local university, all of them with vast experience in questionnaire designing (acknowledged in the manuscript). Their feedback and recommendations were considered with what was suitable for the study.

#### Face validity

The face validity of the survey was also tested by approaching 20 participants (approx. 5% of the total estimated sample) from various hospitals in the country. While answering the questions, participants were asked to orally describe how they felt the questionnaire appeared, was formatted, simple to understand, and was easy to read and interpret. The authors discussed the comments and ideas obtained from the participants before finalizing the tool. Based on the feedback from the respondents, minor changes were made to the questionnaire's final appearance, such as using circle bullet points for answer options and making the title of each section in bold line to make it easier to distinguish each section from the others. These respondents' data were excluded from the final study.

#### Reliability analysis

The pilot study was carried out on 5% of the overall study population. The reliability of the tool was checked by calculating Cronbach's alpha value for each variable as this test is the most commonly used measure of internal consistency reliability [30]. Less than 0.5 was considered to be a sign of low reliability, between 0.5 and 0.7, of moderate reliability, above 0.7, of good reliability, and above 0.8, of excellent reliability [31]. For two of the questionnaire's components—attitude and barriers—the reliability results from the pilot study show low Cronbach's alpha values. Due to the removal of two questions from the attitude section (I think ADR reporting is the responsibility of pharmacists only, It is NOT important to establish ADR monitoring center in every hospital) and two questions from the barriers section (The barriers of ADRs reporting can be due to lack of information provided by the patient, The barriers of ADRs reporting can be due to the belief that reporting is time consuming for pharmacist) after carefully reading the analysis sheet, the values have increased. The reliability of the present study tool was good and significant after did the modifications (α = 0.76; *p* < 0.05). The reliability for each variable was 0.80, 0.83, 0.84, 0.65, 0.93 for knowledge questions, attitude questions, practice questions, barriers statements and suggestions items, respectively. Data from the pilot testing were not included in the main study. The initial original questionnaire was composed of 55 questions and after the modifications, the final questionnaire consisted of 51 questions.

#### Data collection method

To collect data from hospital pharmacists, a web-based online approach was used. The link to the Google survey was distributed individually via email, WhatsApp groups, and social media platforms (Instagram, Facebook, and LinkedIn). The survey's aim, nature, and benefits were all listed on its opening page, which was followed by an option to agree or disagree. The questionnaire page was accessible to those who clicked the agree button after giving their consent and agreement to participate in the study.

#### Data analysis

The data were analyzed using IBM SPSS (Statistical Package for the Social Sciences) Version 28. The data normality distribution was checked using Shapiro–Wilk test, and the outcome revealed that the data were not normally distributed because the *p*-value was less than 0.001. Noncontinuous variables were assessed descriptively and frequency (percentage) and median (IQR) were used for continuous variables. Since the data were not normally distributed, nonparametric tests (Kruskal–Wallis and Mann–Whitney) were used. Post hoc analyses were performed for significant values with more than two groups using the Bonferroni–Dunn correction. A *p*-value of 0.05 was considered statistically significant. The correlation between hospital pharmacists’ knowledge, attitude, practice, qualifications, years of experience, and age was assessed using Spearman’s test. The Spearman's test was used to analyze the correlation between ADR reporting barriers, ADR reporting enhancers, pharmacists' qualifications, years of experience, and age.

## Results

### Demographic characteristics of the study participants

The study questionnaire was sent to 386 pharmacists who work at various hospitals around the UAE. Among them, 342 agreed to complete the questionnaire, resulting in an 88.8% response rate. Lack of time was the primary reported excuse for non-participation. Around 183 (53.5%) of participants were women and 159 (46.5%) were men of age between 20 and 60 years representing 28 different nationalities (Table 2).

**Table 2:** Socio-demographic characteristics of the pharmacists (*n* = 342)

Demographic characteristics	Numbers	Frequency (%)
Gender		
Female	183	53.5
Male	159	46.5
Age		
20–29	139	40.6
30–39	164	48
40–49	33	9.6
≥ 50	6	1.8
Nationality		
Emirati	8	2.3
Non-Emirati:	334	97.7
- Middle East (Saudi Arabia, Kuwait, Bahrain, Oman, Jordan, Syria, Palestine, Lebanon, Iraq, Iran, Yemen)	133	38.9
- Asia (India, Pakistan, Philippines, Bangladesh)	104	30.4
- Africa (Moroccan, Tunisia, Libya, Algeria, Egypt, Sudan, Nigeria, Somalia)	92	26.9
- America and North America (USA, Canada)	3	0.88
- Europe (UK, Cyprus)	2	0.58
Qualification		
Diploma in Pharmacy	10	2.9
Bachelor in Pharmacy	167	48.8
PharmD	63	18.4
Master in Pharmacy	97	28.4
PhD	5	1.5
Country of graduation		
UAE	124	36.3
Other countries:	218	63.7
- Middle East (Saudi Arabia, Kuwait, Oman, Jordan, Syria, Lebanon, Iraq, Iran)	37	10.8
- Asia (India, Pakistan, Philippines, Bangladesh)	101	29.5
- Africa (Libya, Algeria, Egypt, Sudan, Somalia)	72	21.1
- America and North America (USA, Canada)	5	1.46
- Europe (UK, Cyprus)	3	0.88
Current position		
Pharmacist technician	3	0.9
Pharmacist	185	54.1
Clinical pharmacist	50	14.6
Pharmacist in charge	39	11.4
Inpatient pharmacist	31	9.1
Outpatient pharmacist	25	7.3
Pharmacy manager	9	2.6
Years of experiences		
< 5	180	52.6
5–9	107	31.1
10–14	38	11.1
≥ 15	17	5
Type of setting (hospitals)		
Semi-governmental hospitals	32	9.4
Private non-teaching hospitals	240	70.02
Private teaching hospitals	21	6.1
Governmental hospitals	49	14.3
Name of the Emirates		
Abu Dhabi	103	30.1
Dubai	128	37.4
Northern Emirates (Sharjah, Ajman, Ras Al Khaimah, Umm Al Quwain, Fujairah)^a^	111	32.5

^a^Number of participants from each emirate: Sharjah (*n* = 62), Ajman (*n* = 39), Ras Al Khaimah (*n* = 7), Umm Al Quwain (*n* = 2), Fujairah (*n* = 1)*

### Knowledge about PV, ADRs and their reporting

In the questionnaire, nine items were designed to assess the pharmacist’s knowledge of PV, ADRs and their reporting (Table 2). About 93.3% of participants knew the best definition of PV according to WHO definition and 86.8% selected the correct definition of ADR. When asked about the location of international center for adverse drug reaction (ADR) monitoring center, only 134 (39.2%) knew the correct answer. While 221 (64.6%) of pharmacists knew that Naranjo scale is the most common tool used to establish the causality of an ADR. On the other hand, only 151 (44.2%) of respondents knew that ‘Vigibase’ is the WHO online database for reporting ADR (Table 3).

**Table 3:** Knowledge of pharmacists towards PV and ADRs reporting (*n* = 342)

Knowledge questions	Correct *n* (%)	Incorrect *n* (%)
1. What is the best definition of Pharmacovigilance (PV)?	319 (93.3)	23 (6.7)
2. Which of the following defines an adverse drug reaction (ADR) correctly?	297 (86.8)	45 (13.2)
3. The need for hospitalization is required as early as the appearance of:	104 (30.4)	238 (69.6)
4. Seventy-year-old man is taking amiodarone for cardiac arrhythmia and he developed heart block as a side effect. Which of the following matches the type of adverse drug reaction in this patient?	154 (45)	188 (55)
5. Fifteen-year-old boy was given injection of Benzylpenicillin for rheumatic heart disease prophylaxis and developed anaphylaxis as a side effect. Which of the following matches the type of adverse drug reaction in this patient?	206 (60.2)	136 (39.8)
6. A side effect is classified as acute, when it is occurred:	105 (30.7)	237 (69.3)
7. The international center for adverse drug reaction (ADR) monitoring is located in:	134 (39.2)	208 (60.8)
8. Which of the following tool is most commonly used to establish the causality of an adverse drug reaction (ADR)?	221 (64.6)	121 (35.4)
9. Which of the following is the “WHO online databases” for reporting adverse drug reactions (ADRs)?	151 (44.2)	191 (55.8)

The overall median knowledge score (IQR) was 5 (3–7) and the maximum possible knowledge score was 9. Using the Mann–Whitney test, there are no statistically significant differences among gender on the knowledge of pharmacovigilance and ADR reporting (*p*-value = 0.778), with a similar median (IQR) total score of 5 (3–7) for both males and females. When using the Kruskal–Wallis test, it was shown that the knowledge levels within the various qualification groups varied, with a high statistical significance (*p*-value < 0.001). The median is as follows: Diploma in pharmacy 4.5 (3–6.25), Bachelor in pharmacy 4 (3–6), PharmD 6 (4–7), Master in Pharmacy 5 (4–7), Ph.D. 5 (4–6.5) (Table 4). Further post hoc analysis showed that clinical pharmacists and inpatient pharmacists are more knowledgeable in pharmacovigilance and ADR reporting than pharmacists (*p*-value < 0.001 using the Bonferroni test). The test also revealed that hospital pharmacists with years of experience between 10 and 14 have more knowledge about pharmacovigilance and ADR reporting than hospital pharmacists with less than 5 years of experience (*p*-value < 0.001). Hospital pharmacists holding Pharm D certificates or Master’s degrees in pharmacy are better knowledgeable regarding pharmacovigilance and ADR reporting than hospital pharmacists holding bachelor's degrees only (*p*-value = 0.001, *p*-value = 0.008, respectively). It seems that knowledge of PV is directly affected by the level of education and years of experience.

**Table 4:** Pharmacists’ knowledge towards PV and ADR reporting vs sociodemographic characteristics

Demographic characteristics	Median knowledge score (IQR)	*p*-value
Gender		
Female	5 (3–7)	0.778^a^
Male	5 (3–7)	
Age		
20–29	4 (3–6)	0.103^b^
30–39	5 (4–7)	
40–49	5 (4–6.5)	
≥ 50	6 (2.75–8)	
Nationality		
Emirati	5 (3.25–6)	0.448^b^
Non-Emirati:		
- Middle East (Saudi Arabia, Kuwait, Bahrain, Oman, Jordan, Syria, Palestine, Lebanon, Iraq, Iran, Yemen)	4 (3–7)	
- Asia (India, Pakistan, Philippines, Bangladesh)	5 (4–7)	
- Africa (Moroccan, Tunisia, Libya, Algeria, Egypt, Sudan, Nigeria, Somalia)	5 (3–7)	
- America and North America (USA, Canada)^c^	–	
- Europe (UK, Cyprus)^c^	–	
Qualification		
Diploma in Pharmacy	4.5 (3–6.25)	**< 0.001**^b,*^
Bachelor in Pharmacy	4 (3–6)	
PharmD	6 (4–7)	
Master of Science in Pharmacy	5 (4–7)	
PhD	5 (4–6.5)	
Country of graduation		
UAE	5 (3–7)	0.077^b^
Other countries:		
- Middle East (Saudi Arabia, Kuwait, Oman, Jordan, Syria, Lebanon, Iraq, Iran)	4 (3–6)	
- Asia (India, Pakistan, Philippines, Bangladesh)	5 (4–7)	
- Africa (Libya, Algeria, Egypt, Sudan, Somalia)	5 (3.25–7)	
- America and North America (USA, Canada)	3 (3–4.5)	
- Europe (UK, Cyprus)^c^	–	
Current position		
Pharmacist technician^c^	–	** < 0.001**^b,*^
Pharmacist	4 (3–6)	
Clinical pharmacist	6 (5–7)	
Pharmacist in charge	5 (3–7)	
Inpatient pharmacist	7 (4–8)	
Outpatient pharmacist	5 (3–6)	
Pharmacy manager	5 (4–5.5)	
Years of experiences		
< 5	4 (3–6)	** < 0.001**^b,*^
5–9	6 (4–7)	
10–14	6.5 (4–8)	
≥ 15	5 (4–6.5)	
Type of setting (hospitals)		
Semi-governmental hospitals	5 (3–7)	0.083^b^
Private non-teaching hospitals	4 (3–7)	
Private teaching hospitals	6 (4–7)	
Governmental hospitals	5 (3–7.5)	
Name of the emirates		
Abu Dhabi	5 (3–6)	0.452^b^
Dubai	5 (3–7)	
Northern Emirates (Sharjah, Ajman, Ras Al Khaimah, Umm Al Quwain, Fujairah)	5 (3–6)	

Bold values indicate statistical significance

*IQR* interquartile range

^a^Mann–Whitney test

^b^Kruskal–Wallis test

^c^The total responses may not sum up to 100% in few cases due to low intervals

^*^Significant (*p* < 0.05)

### Attitudes about ADRs and their reporting

Regarding attitude about reporting ADRs, most hospital pharmacists who participated in the study (90.6%) are willing to spare adequate time to review patients’ ADR reports with their management. Among the participants, 238 (69.6%) believe that pharmacists who report ADRs should be rewarded. Only 26 (7.6%) of those surveyed support the idea that patients should not directly disclose any ADR they encounter (Table 5).

**Table 5:** Attitude towards ADR reporting among hospital pharmacists (*n* = 342)

Attitude items	Level of agreement, *n* (%)					Median (IQR) scores
	Strongly Disagree (1)	Disagree (2)	Neutral (3)	Agree (4)	Strongly Agree (5)	
1. I am willing to spend enough time to discuss patient adverse drug reaction (ADR) on regular basis with my manager	11 (3.2)	5 (1.5)	16 (4.7)	198 (57.9)	112 (32.7)	4 (4–5)
2. There should be an incentive for pharmacists who are reporting ADR	19 (5.6)	27 (7.9)	58 (17)	133 (38.9)	105 (30.7)	4 (3–5)
3. Patient should NOT allow to report ADR^a^	122 (35.7)	169 (49.4)	25 (7.3)	18 (5.3)	8 (2.3)	2 (1–2)
4. I believe that ADR reporting should be made mandatory for practicing pharmacists	16 (4.7)	20 (5.8)	51 (14.9)	144 (42.1)	111 (32.5)	4 (3–5)
5. It is important to report ADRs in order to answer the questions that may arise in my practice	5 (1.5)	2 (0.6)	35 (10.2)	190 (55.6)	110 (32.2)	4 (4–5)
6. Reporting of ADRs is important to show patients that their concerns are taken seriously	9 (2.6)	7 (2)	34 (9.9)	171 (50)	121 (35.4)	4 (4–5)

*IQR* interquartile range

^a^Scoring for item 3 is reversed, as the statement was negatively worded

The maximum possible attitude score was 30, and the overall median (IQR) attitude score was 22 (20–24). The positions of hospital pharmacists and their years of experience with their attitudes toward pharmacovigilance and ADR reporting are significantly correlated (*p*-values 0.004, 0.002, respectively). Table 6 contains further information.

**Table 6:** Pharmacists’ attitude towards PV and ADR reporting vs sociodemographic characteristics

Demographic characteristics	Median knowledge score (IQR)	*p*-value
Gender		
Female	22 (20–24)	0.815^a^
Male	22 (21–24)	
Age		
20–29	22 (21–24)	0.540^b^
30–39	22 (20–24)	
40–49	23 (19–25)	
≥ 50	24 (20–26)	
Nationality		
Emirati	22 (20–23)	0.679^b^
Non-Emirati:		
- Middle East (Saudi Arabia, Kuwait, Bahrain, Oman, Jordan, Syria, Palestine, Lebanon, Iraq, Iran, Yemen)	22 (20–24)	
- Asia (India, Pakistan, Philippines, Bangladesh)	22 (21–24)	
- Africa (Moroccan, Tunisia, Libya, Algeria, Egypt, Sudan, Nigeria, Somalia)	22 (20–24.75)	
- America and North America (USA, Canada)^c^	–	
- Europe (UK, Cyprus)^c^	–	
Qualification		
Diploma in Pharmacy	22.5 (18.75–23.25)	0.059^b^
Bachelor in Pharmacy	22 (20–24)	
PharmD	23 (21–25)	
Master in Pharmacy	23 (21–24.5)	
PhD	25 (23–26.5)	
Country of graduation		
UAE	23 (21–25)	0.424^b^
Other countries:		
- Middle East (Saudi Arabia, Kuwait, Oman, Jordan, Syria, Lebanon, Iraq, Iran)	22 (20–24)	
- Asia (India, Pakistan, Philippines, Bangladesh)	22 (21–24)	
- Africa (Libya, Algeria, Egypt, Sudan, Somalia)	22 (20–24)	
- America and North America (USA, Canada)	21 (20–23.5)	
- Europe (UK, Cyprus)^c^	–	
Current position		
Pharmacist technician^c^	–	**0.004**^b,*^
Pharmacist	22 (20–24)	
Clinical pharmacist	23 (21–25)	
Pharmacist in Charge	23 (20–25)	
Inpatient pharmacist	24 (22–25)	
Outpatient pharmacist	21 (20–22)	
Pharmacy manager	22 (17.5–23.5)	
Years of experiences		
< 5	22 (20–24)	**0.002**^b,*^
5–9	23 (21–24)	
10–14	23 (21.75–26)	
≥ 15	24 (19.5–25.5)	
Type of setting (hospitals)		
Semi-governmental hospitals	23 (21–26)	0.183^b^
Private non-teaching hospitals	22 (20–24)	
Private teaching hospitals	23 (21–25)	
Governmental hospitals	22 (20–25)	
Name of the emirates		
Abu Dhabi	23 (20–25)	0.187^b^
Dubai	23 (20–24.75)	
Northern Emirates (Sharjah, Ajman, Ras Al Khaimah, Umm Al Quwain, Fujairah)	22 (21–23)	

Bold values indicate statistical significance

*IQR* interquartile range

^a^Mann–Whitney test

^b^Kruskal–Wallis test

^c^The total responses may not sum up to 100% in few cases due to low intervals

^*^Significant (*p* < 0.05)

### Practices and barriers to ADRs and their reporting

Based on this research, 243 (71.1%) observed ADRs during the previous year. Of those, 182 (53.2%) reported the ADR to the concerned bodies (Table 7). The number of reported cases is between 1 and 5 (40.9%). Of participants, 254 (74.3%) reported that their workplace provides information regarding the procedure of ADR reporting. One hundred fifty-three hospital pharmacists (44.74%) mentioned that they received training about ADR reporting. Table 8 shows the median scores (IQR) for each practice-related question.

**Table 7:** Reporting departments (*n* = 177^a^)

Reporting institution	Numbers	Frequency (%)
Ministry of Health (MOH)	59	17.3
Dubai Healthcare Authority (DHA)	42	12.3
Both MOH and DHA	27	7.9
Drug manufacturer	31	9.1
Physician	1	0.3
All of the above	2	0.6
Hospital management	6	1.8
Clinical pharmacist in hospital	2	0.6
Quality department	7	2

^a^The number of pharmacists who did reporting during last year

**Table 8:** Pharmacovigilance (PV) and adverse drug reaction (ADR) reporting practices (*n* = 342)

Practice questions	Yes *n* (%)	No *n* (%)	Median (IQR) scores
Have you observed any adverse drug reactions in your practice in the past one year?	243 (71.1)	99 (29)	1 (1–2)
Have you ever reported any adverse drug reactions in the past one year?	177 (52)	165 (48.3)	1 (1–2)
Does your workplace provide information regarding the procedure of reporting adverse drug reactions?	254 (74.3)	88 (25.7)	1 (1–2)
Did you take any training in adverse drug reactions reporting at your work place?	153 (44.7)	189 (55.3)	2 (1–2)
Does your workplace encourage you to report an adverse drug reaction?	279 (81.6)	63 (18.4)	1 (1–1)
Is adverse drug reaction reporting mandatory at your current work place?	195 (57)	147 (43)	1 (1–2)

The overall median (IQR) practice score was 17.5 (11–21) and the maximum practice score was 24. Significant correlations exist between hospital pharmacists' positions, years of experience, and practices related to pharmacovigilance and ADR reporting (*p*-values 0.004, 0.002, respectively) (Table 9).

**Table 9:** Pharmacists’ practice towards PV and ADR reporting vs sociodemographic characteristics

Demographic characteristics	Median knowledge score (IQR)	*p*-value
Gender		
Female	18 (11–21)	0.452^a^
Male	16 (11–21)	
Age		
20–29	19 (12–21)	0.540^b^
30–39	13.5 (11–20.75)	
40–49	15 (11–19)	
≥ 50	13.5 (11–19.25)	
Nationality		
Emirati	13.5 (10–21)	0.679^b^
Non-Emirati:		
- Middle East (Saudi Arabia, Kuwait, Bahrain, Oman, Jordan, Syria, Palestine, Lebanon, Iraq, Iran, Yemen)	17 (11–20)	
- Asia (India, Pakistan, Philippines, Bangladesh)	15 (11–20)	
- Africa (Moroccan, Tunisia, Libya, Algeria, Egypt, Sudan, Nigeria, Somalia)	19 (12–21)	
- America and North America (USA, Canada)^c^	–	
- Europe (UK, Cyprus)^c^	–	
Qualification		
Diploma in Pharmacy	18.5 (12.75–19.5)	0.059^b^
Bachelor in Pharmacy	19 (12–21)	
PharmD	13 (11–20)	
Master in Pharmacy	15 (11–20)	
PhD	14 (10.5–18.5)	
Country of graduation		
UAE	14.5 (11–20)	0.424^b^
Other countries:		
- Middle East (Saudi Arabia, Kuwait, Oman, Jordan, Syria, Lebanon, Iraq, Iran)	17 (11.5–21.5)	
- Asia (India, Pakistan, Philippines, Bangladesh)	15 (11–20)	
- Africa (Libya, Algeria, Egypt, Sudan, Somalia)	19 (12–21)	
- America and North America (USA, Canada)	22 (15–22)	
- Europe (UK, Cyprus)^c^	–	
Current position		
Pharmacist technician^c^	–	**0.004**^b,*^
Pharmacist	19 (12.5–21)	
Clinical pharmacist	12 (11–17.25)	
Pharmacist in charge	15 (11–20)	
Inpatient pharmacist	13 (11–19)	
Outpatient pharmacist	13 (11.5–21)	
Pharmacy manager	13 (11–19)	
Years of experiences		
< 5	19 (12–21)	**0.002**^b,*^
5–9	13 (11–20)	
10–14	13 (11–18.25)	
≥ 15	11 (10.5–16.5)	
Type of setting (hospitals)		
Semi-governmental hospitals	17.5 (11–21)	0.183^b^
Private non-teaching hospitals	18.5 (12–21)	
Private teaching hospitals	11 (10–13)	
Governmental hospitals	13 (10.5–19)	
Name of the emirates		
Abu Dhabi	16 (12–20)	0.187^b^
Dubai	18 (11–20)	
Northern Emirates (Sharjah, Ajman, Ras Al Khaimah, Umm Al Quwain, Fujairah)	19 (11–22)	

Bold values indicate statistical significance

*IQR* interquartile range

^a^Mann–Whitney test

^b^Kruskal–Wallis test

^c^The total responses may not sum up to 100% in few cases due to low intervals

^*^Significant (*p* < 0.05)

The Bonferroni test was utilized in order to assess the correlations between the variables which are statistically significant. The findings demonstrated that hospital pharmacists who are in a position of clinical pharmacists engaged in pharmacovigilance more frequently than those who are in a position of pharmacist only (*p*-value =  < 0.001). The same is true for inpatient pharmacists; they report more ADRs than those who work as sole pharmacists (*p*-value = 0.018).

### The barriers to implement PV and ADR reporting

The difficulties hospital pharmacists have in adopting PV and ADR reporting are listed in Table 10. Approximately half of the participants strongly agreed or agreed that seven of the eight issues mentioned in the study were obstacles to implement PV. Considering ADR reporting is not a part of pharmacist’s job was perceived as a barrier by 39 (11.4%) participants, while 281 (82.2%) disagreed or strongly disagreed with this statement. More than half of the pharmacists (57%) agreed that “Lack of awareness about the reporting process” is a barrier with another 20.2% strongly agreeing with that hindrance. Another perceived barrier is “All serious ADRs are already detected before registration of drug” which took the highest disagreement with (45.9%) and (18.4%) strongly disagreement.

**Table 10:** The barriers to implement PV and ADR reporting (*n* = 342)

Statement	Level of agreement, *n* (%)					Median (IQR) scores
	Strongly Disagree (1)	Disagree (2)	Neutral (3)	Agree (4)	Strongly Agree (5)	
1. It is not a part of pharmacist’s job	159 (46.5)	122 (35.7)	22 (6.4)	27 (7.9)	12 (3.5)	2 (1–2)
2. Lack of awareness about the reporting process	7 (2.1)	33 (9.7)	38 (11.1)	195 (57)	69 (20.2)	4 (4–4)
3. All serious ADRs are already detected before registration of drug	63 (18.4)	157 (45.9)	65 (19)	45 (13.2)	12 (3.5)	2 (2–3)
4. Fear of consequences after reporting (i.e., legal actions or reduced patient’s confidence)	12 (3.5)	51 (14.9)	63 (18.4)	169 (49.4)	47 (13.7)	4 (3–4)
5. Lack of awareness of the existence of a national ADR reporting system	9 (2.6)	32 (9.4)	30 (8.8)	176 (51.5)	95 (27.8)	4 (4–5)
6. Pharmacovigilance topic not included in pharmacy curriculum	27 (7.9)	113 (33)	61 (17.8)	93 (27.2)	48 (14)	3 (2–4)
7. Lack of proper training on ADR reporting	12 (3.5)	30 (8.8)	28 (8.2)	169 (49.4)	103 (30.1)	4 (4–5)
8. Difficulty in deciding whether ADR had occurred or not	25 (7.3)	71 (20.8)	70 (20.5)	129 (37.7)	47 (13.7)	4 (2–4)

The overall median (IQR) scores to barriers was 26 (23–29) and the maximum barrier score was 40. The Kruskal–Wallis test revealed a statistically significant association between the pharmacists' positions and their practice of pharmacovigilance and ADR reporting (*p*-value: < 0.001). Additionally, the practice of ADR reporting also is statistically significant across different emirates (*p*-value: < 0.001) (Table 11).

**Table 11:** The barriers to implement PV and ADR reporting vs sociodemographic characteristics

Demographic characteristics	Median knowledge score (IQR)	*p*-value
Gender		
Female	27 (23–29)	0.447^a^
Male	26 (23–29)	
Age		
20–29	26 (23–29)	0.192^b^
30–39	27 (24–29)	
40–49	26 (21.5–29)	
≥ 50	23.5 (21–27.5)	
Nationality		
Emirati	29 (27.25–30.75)	0.170^b^
Non-Emirati:		
- Middle East (Saudi Arabia, Kuwait, Bahrain, Oman, Jordan, Syria, Palestine, Lebanon, Iraq, Iran, Yemen)	26 (23–28.5)	
- Asia (India, Pakistan, Philippines, Bangladesh)	26 (23–29)	
- Africa (Moroccan, Tunisia, Libya, Algeria, Egypt, Sudan, Nigeria, Somalia)	27 (23.25–29)	
- America and North America (USA, Canada)^c^	–	
- Europe (UK, Cyprus)^c^	–	
Qualification		
Diploma in Pharmacy	24 (21.5–29.75)	0.199^b^
Bachelor in Pharmacy	27 (23–30)	
PharmD	26 (23–28)	
Master in Pharmacy	26 (24–28)	
PhD	22 (20.5–25)	
Country of graduation		
UAE	26 (23–28.75)	0.501^b^
Other countries:		
- Middle East (Saudi Arabia, Kuwait, Oman, Jordan, Syria, Lebanon, Iraq, Iran)	26 (23.5–28.5)	
- Asia (India, Pakistan, Philippines, Bangladesh)	26 (23–29)	
- Africa (Libya, Algeria, Egypt, Sudan, Somalia)	27 (25–29)	
- America and North America (USA, Canada)	29 (26.5–30)	
- Europe (UK, Cyprus)^c^	–	
Current position		
Pharmacist technician^c^	–	** < 0.001**^b,*^
Pharmacist	27 (23–29)	
Clinical pharmacist	25 (22–27)	
Pharmacist in charge	27 (24–30)	
Inpatient pharmacist	25 (23–27)	
Outpatient pharmacist	29 (25–31)	
Pharmacy manager	24 (20–25)	
Years of experiences		
< 5	27 (23.25–29)	0.008^b^
5–9	26 (24–29)	
10–14	25.5 (22.75–27)	
≥ 15	23 (18–26.5)	
Type of setting (hospitals)		
Semi-governmental hospitals	28 (26–29)	0.193^b^
Private non-teaching hospitals	26 (23–29)	
Private teaching hospitals	25 (23–27)	
Governmental hospitals	26 (22–29)	
Name of the emirates		
Abu Dhabi	25 (22–28)	** < 0.001**^b,*^
Dubai	26 (23–28)	
Northern Emirates (Sharjah, Ajman, Ras Al Khaimah, Umm Al Quwain, Fujairah)	28 (26–30)	

Bold values indicate statistical significance

*IQR* interquartile range

^a^Mann–Whitney test

^b^Kruskal–Wallis test

^c^The total responses may not sum up to 100% in few cases due to low intervals

^*^Significant (*p* < 0.05)

According to the Bonferroni test, hospital pharmacists who serve as outpatient pharmacists face more obstacles while reporting adverse drug reactions (ADRs) than clinical pharmacists (*p*-value = 0.015). The test also showed that reporting ADRs presents more challenges for outpatient pharmacists than for pharmacists in charge (*p*-value = 0.024). The test’s findings also indicated that there are more obstacles in the northern emirates than in Abu Dhabi and Dubai (*p*-value =  < 0.001 for both the northern emirates with Abu Dhabi and the northern emirates with Dubai).

### Factors that would encourage pharmacists to report ADRs and implement PV system

The most crucial variables that may enhance reporting are by encouraging all healthcare professionals to report ADRs (63.5%). Conducting workshops and ongoing education for pharmacists comes in second (60.5%). Table 12 lists additional factors that the study recommended to encourage hospital pharmacists to report ADRs. Only (17.5%) believed that patients can directly do reporting to the national pharmacovigilance center.

**Table 12:** Factors that would encourage pharmacists to report an ADRs and implement PV system (*n* = 342)

Statement	Level of agreement, *n* (%)					Median (IQR) scores
	Strongly Disagree (1)	Disagree (2)	Neutral (3)	Agree (4)	Strongly Agree (5)	
1. There should be incentives for the pharmacist who perform the reporting	9 (2.6)	34 (9.9)	62 (18.1)	143 (41.8)	94 (27.5)	4 (3–5)
2. Availability of ADR reporting center in each hospital will enhance PV activity	1 (0.3)	5 (1.5)	20 (5.9)	167 (48.8)	149 (43.6)	4 (4–5)
3. Direct ADR reporting by patients to national PV center	16 (4.7)	52 (15.2)	92 (26.9)	122 (35.7)	60 (17.5)	4 (3–4)
4. Proper training regarding the procedure of reporting ADRs will encourage reporting by pharmacists	0	4 (1.2)	12 (3.5)	176 (51.5)	150 (43.9)	4 (4–5)
5. Legal protection should be provided to the pharmacists by their workplace or by the relevant authority if they have dispensed the medication causing ADR	5 (1.5)	9 (2.6)	29 (8.5)	153 (44.7)	146 (42.7)	4 (4–5)
6. Continuous education and workshops for pharmacists	3 (0.9)	2 (0.6)	13 (3.8)	117 (34.2)	207 (60.5)	5 (4–5)
7. Encourage all health professionals to report	2 (0.6)	2 (0.6)	12 (3.5)	109 (31.9)	217 (63.5)	5 (4–5)
8. Ease of access to ADR forms	2 (0.6)	9 (2.6)	13 (3.8)	114 (33.3)	204 (59.7)	5 (4–5)
9. Using information technology in facilitating ADR reporting in the country	2 (0.6)	4 (1.2)	9 (2.6)	130 (38)	197 (57.6)	5 (4–5)
10. PV should be taught in the pharmacy curriculum	6 (1.8)	3 (0.9)	24 (7)	121 (35.4)	188 (55)	5 (4–5)
11. Difficult to decide whether or not an ADR has occurred	16 (4.7)	46 (13.5)	77 (22.5)	101 (29.5)	102 (29.8)	4 (3–5)

The overall median (IQR) scores to factors that may enhance pharmacovigilance and ADR reporting was 47 (43–59) and the maximum barrier score was 55. The Kruskal–Wallis test demonstrated that pharmacists’ position can significantly improve ADR reporting (Table 13).

**Table 13:** The suggestions to implement PV and ADR reporting vs sociodemographic characteristics

Demographic characteristics	Median knowledge score (IQR)	*p*-value
Gender		
Female	47 (43–50)	0.879^a^
Male	47 (44–50)	
Age		
20–29	47 (43–50)	0.626^b^
30–39	48 (44–51)	
40–49	47 (40.5–50)	
≥ 50	49 (42.5–52.25)	
Nationality		
Emirati	44 (43.25–49.5)	0.520^b^
Non-Emirati:		
- Middle East (Saudi Arabia, Kuwait, Bahrain, Oman, Jordan, Syria, Palestine, Lebanon, Iraq, Iran, Yemen)	47 (44–51)	
- Asia (India, Pakistan, Philippines, Bangladesh)	46 (43–50)	
- Africa (Moroccan, Tunisia, Libya, Algeria, Egypt, Sudan, Nigeria, Somalia)	47 (44–50)	
- America and North America (USA, Canada)^c^	–	
- Europe (UK, Cyprus)^c^	–	
Qualification		
Diploma in Pharmacy	42 (37.5–46.75)	**0.048**^**b**^
Bachelor in Pharmacy	47 (43–50)	
PharmD	47 (42–50)	
Master in Pharmacy	48 (45–51)	
PhD	45 (42.5–53.5)	
Country of graduation		
UAE	47 (43–50)	0.453^b^
Other countries:		
- Middle East (Saudi Arabia, Kuwait, Oman, Jordan, Syria, Lebanon, Iraq, Iran)	49 (45–52)	
- Asia (India, Pakistan, Philippines, Bangladesh)	46 (43–50)	
- Africa (Libya, Algeria, Egypt, Sudan, Somalia)	47 (44–50)	
- America and North America (USA, Canada)	49 (44–53)	
- Europe (UK, Cyprus)^c^	–	
Current position		
Pharmacist technician^c^	–	** < 0.001**^b,*^
Pharmacist	46 (43–50)	
Clinical pharmacist	46 (42.75–51)	
Pharmacist in charge	49 (44–51)	
Inpatient pharmacist	47 (44–50)	
Outpatient pharmacist	50 (48.5–52)	
Pharmacy manager	43 (31–45)	
Years of experiences		
< 5	47 (44–50)	0.373^b^
5–9	48 (43–50)	
10–14	48 (43.75–51)	
≥ 15	44 (39–50.5)	
Type of setting (hospitals)		
Semi-governmental hospitals	47.5 (43–52)	0.505^b^
Private non-teaching hospitals	47 (43–50)	
Private teaching hospitals	48 (43.5–51)	
Governmental hospitals	48 (44–51)	
Name of the emirates		
Abu Dhabi	47 (43–50)	**0.027**^**b**^
Dubai	46.5 (42.25–49.75)	
Northern Emirates (Sharjah, Ajman, Ras Al Khaimah, Umm Al Quwain, Fujairah)	49 (44–51)	

Bold values indicate statistical significance

*IQR* interquartile range

^a^Mann–Whitney test

^b^Kruskal–Wallis test

^c^The total responses may not sum up to 100% in few cases due to low intervals

^*^Significant (*p* < 0.05)

### Correlations between items of hospital pharmacists towards PV and ADR reporting

The correlation test showed a positive relationship between hospital pharmacists’ knowledge and their qualifications, years of experience, and age. On the other hand, there was a negative correlation between hospital pharmacists’ practice of reporting ADRs and their qualifications, age, and years of experience (Additional file 1).

## Discussion

The purpose of this study was to assess hospital pharmacists' knowledge, attitude, and practices regarding ADR reporting, as well as to identify the major barriers that prevent the implementation of a PV system in the UAE and the factors that may help enhance ADR reporting. The results indicate that although hospital pharmacists have very positive attitudes toward reporting ADRs, there is still a low level of reporting among them. This can be the result of insufficient knowledge related to PV and ADRs such as what to report, where to report, and when to report.

The study's findings showed that the majority of pharmacists were well-versed in the definitions of PV and ADRs. This result is consistent with other studies in Kuwait [13], Sudan [14], India [15], Brazil [10], Pakistan [6, 16], Saudi Arabia [32–34] and UAE [17]. In opposition to these studies, other previous studies conducted in Ethiopia [11], India [1], and Saudi Arabia [12, 35] indicated that pharmacists who participated in these studies had a low level of knowledge about PV and ADRs terminology. Training sessions and continuing medical education conferences can be arranged, and the subject of PV can be incorporated into undergraduate curricula to increase the level of understanding among pharmacists.

Additionally, the findings indicated that participants were unaware of the locations of both national and worldwide ADR monitoring centers as well as the WHO's online ADR reporting database. This is an important finding that is undoubtedly related to the current underreporting of ADRs. This finding is in line with studies in Saudi Arabia [18, 32], Nigeria [19], Pakistan [6], UAE [9], Kuwait [13], Syria [20] and Jordan [12] where underreporting of ADRs are due to being unaware of a national ADR reporting center or the procedures for reporting ADRs. All of this shows that, in addition to creating a national PV program and ensuring that it achieves its objectives, it is crucial to disseminate information and give end users proper training. As other studies showed the impact of training to improve the understanding of health professionals on the reporting scheme [1, 11, 36].

Participants' awareness of PV and ADR reporting was unaffected by their gender; however, it was affected by their educational level. Pharmacists lack expertise in PV and ADR reporting contrary to clinical pharmacists and inpatient pharmacists. This might be due to the fact that clinical pharmacists studied PV, medication errors, drug-related problems, ADR detection, reporting, and causality assessment for their master's degree, and, in the case of inpatient pharmacists, they have extensive patient-care experience. This is comparable to the findings of a study done in Sudan, which showed no statistically significant difference in the mean knowledge score between the genders [12], and with the findings of a study conducted in India, which demonstrated that pharmacists' educational background influences their level of knowledge about reporting ADRs [32].

Years of experience are another factor that might have an impact on knowledge level. According to the current study, participants' years of experience have a big impact on their knowledge level. Hospital pharmacists with fewer than five years of experience lack the expertise that pharmacists with 10–14 years of experience have about pharmacovigilance and ADR reporting. This result is similar to the earlier study conducted among community pharmacists in Sudan [14].

Regarding the attitude of hospital pharmacists, our findings indicated a positive attitude towards ADRs reporting. Similar result was also observed by studies conducted in Iraq [37], Saudi Arabia [38] and Kuwait [13]. A study conducted in New Zealand, however, found that the participants had a negative response to ADR reporting [39]. More than half of the participants in our study said that pharmacists who report ADRs should be rewarded, and the majority are willing to spend enough time discussing patient ADR reports with their management. Most people also thought that reporting ADRs was a requirement of their profession. Similar research with pharmacists from various nations has confirmed this observation, agreeing that reporting ADRs is a professional duty [12, 13, 40].

Other key findings of the primary data are that although more than 70% of the participants observed ADRs during the previous year, only half of them reported ADRs. This underreporting may be the result of poor time management, lack of awareness of reporting process, difficulties to access ADRs report, and poor communication between patients and pharmacists. The reported figure in this survey may be impacted by the fact that there is no legal requirement for some hospitals to record ADRs. Similarly, studies in different countries also revealed a low reporting rate: Qatar [21], Istanbul [41], Jordan [12], KSA [18], and Northern China [36]. The opposite was observed in Sweden, where 60% of healthcare providers report ADRs as a result of the greater comprehension of the reporting system and strong reporting facilitation by pertinent institutions in Sweden [11].

Other elements identified in this study that influence the hospital pharmacists’ ability to perform ADRs reporting are their position and years of experience. Clinical pharmacists engaged in pharmacovigilance more frequently than those who are in a position of pharmacist only and the same is true for inpatient pharmacists. This can be explained by the fact that clinical pharmacists and inpatient pharmacists are more likely than pharmacists to get insight into the effects of ADRs. This outcome is compatible with other research conducted in Ethiopia [11].

Hospital pharmacists who participated in the survey noted a variety of obstacles to reporting ADRs in the UAE. Only a small percentage of pharmacists (11.4%) say that reporting ADRs is not part of their duties. This finding is consistent with research from Ethiopia [11] and Sweden [42] in which the majority of healthcare workers thought that reporting ADRs was a responsibility of theirs. Participants in Iran [43] reported this disparity, stating that they thought pharmaceutical corporations and legal medical authorities were responsible for reporting ADRs. This suggested that healthcare personnel had a proper understanding of their responsibilities to report ADRs.

The lack of information about the reporting process was cited by more than half of the pharmacists as a barrier. This was also documented by pharmacists in other countries as a barrier [12, 13, 40, 44]. In light of this, pharmacists underlined the importance of educating HCP about the process of reporting ADR as several studies proofed the benefit of educational intervention on the KAP of HCP in ADRs reporting [1, 45–47]. Some participants worry about the repercussions of reporting from their management. This was also reported in Kuwait [13], Saudi Arabia [38] and Iraq [37] as an obstacle. For that, awareness about ADRs reporting should be at the level of HCP and hospital administrators. On the other hand, fewer participants in Jordan [12] and Syria [20] see legal culpability as a barrier. Other obstacles mentioned in this poll include difficulties to determine whether ADR has occurred or not believing that only safe modifications released in the market. Similar restrictions were identified in earlier researches [12, 21, 25, 40, 44, 48]. When reporting ADRs, hospital pharmacists who work as outpatient pharmacists have more challenges than clinical pharmacists. This is due to the fact that dealing with outpatients necessitates asking them many questions in contrast to dealing with inpatients, where patients are constantly being monitored by the healthcare staff and any ADR might be noted. The test's results also showed that the northern emirates had more impediments than Abu Dhabi and Dubai.

The study also emphasized a few elements that could help improve ADR reporting. The findings suggested that there should be incentives for the pharmacist who perform the reporting. Additional research supported this conclusion [12, 13]. Another approach is to conduct workshops and ongoing education for pharmacists. This approach was also proposed by studies conducted in Ethiopia [11], India [1], and Egypt [36]. The ADR reported center availability in each hospital is expected to enhance PV activity. This finding is similar to what other studies found in Saudi Arabia [38] and India [49]. It is recommended to provide legal protection to the pharmacists by relevant authorities in case if any of the medicines dispensed by them have caused an ARD. Ease of access to ADR forms online also could enhance ADR reporting and this result is similar to one conducted in Kuwait where pharmacists would prefer using an email or a web-based reporting system [13]. In contrast, a poll conducted in Jordan found that pharmacists preferred verbal communication with a medication company representative, phone calls, and paper-based forms as reporting techniques over the Internet [12].

## Strengths and limitations of the study

This is the first study to assess the pharmacists’ knowledge, attitude, and practice, of PV in the whole UAE and the factors that influence reporting in hospitals. Besides this strength, the study also has some limitations. First, participants might have shied away from participating because they felt uneasy answering because they did not have enough knowledge about PV and ADRs. If that is the case, it is possible that the questionnaire was only answered by people who had sufficient knowledge of PV and ADRs, which may have impacted the study's findings. Secondly, since the survey was only given to hospital-based pharmacists, it is still unclear whether the findings apply to pharmacists who work in other contexts, such as polyclinics and community pharmacies. Finally, there is also a possibility that the responses could be influenced by the Dunning–Kruger effect.

## Recommendations

Results showed that because pharmacists were unsure about where and how to report ADRs, many did not do so. This necessitates the development of interventional educational initiatives that have been proven to successfully raise international awareness of ADR reporting [45, 50, 51]. Collaboration between academic institutions and health authorities is essential for achieving these objectives. Academic institutions can provide specific training interventions and related ADR reporting systems that are suited to the pharmacy workplace, in accordance with the Health Authorities regulations. To encourage ADR reporting and enhance PV practices in the UAE, it is essential that the Health Authorities provide clear standards and mechanisms to support this attitude.

## Conclusion

The findings of this study demonstrated that despite hospital pharmacists' high knowledge and positive attitudes toward PV and ADR reporting, there is still underreporting of ADRs. The results point to the need for pharmacists, other HCPs, and administrators to get formal, customized training on a regular basis with the goals of clearly identifying PV and ADRs, ADR reporting criteria and deadlines, and where and how to report them. The knowledge and practice gaps between hospital pharmacists regarding the reporting of ADRs may be filled by this educational intervention. The educational program may include presentations, workshops, and small group discussions and it could be delivered by mail, newsletters, reminders, advertisements, and continuous education programs. Future pharmacists should also be trained in pharmacovigilance and ADR reporting procedures.

## Supplementary Information

**Additional file 1:** Correlations between items of hospital pharmacists towards PV & ADR reporting.

## Data Availability

The data that support the findings of this study are available from the first author, upon reasonable request.
