# Supplementary material for: A national survey of knowledge, attitude, practice, and barriers towards pharmacovigilance and adverse drug reaction reporting among hospital pharmacy practitioners in the United Arab Emirates
Source: J Pharm Policy Pract. 2023 Jul 18;16:92. doi: 10.1186/s40545-023-00593-6 (PMC10355021; doi:10.1186/s40545-023-00593-6)
Supplement: Supplementary file 1 — Additional file 1: Correlations between items of hospital pharmacists towards PV & ADR reporting. [file 40545_2023_593_MOESM1_ESM.docx]

**Additional file 1: Correlations between items of hospital pharmacists towards PV & ADR reporting**

| **Item (Spearman correlation)** | **Correlation coefficient (r)** | **p-value** |
| --- | --- | --- |
| **Knowledge** | | |
| ***Knowledge with Qualifications*** |  |  |
| Which of the following defines an Adverse Drug Reaction (ADR) correctly? | 0.142 | 0.008* |
| The need for hospitalization is required as early as the appearance of: | 0.123 | 0.022* |
| The international center for Adverse Drug Reaction (ADR) monitoring is located in: | 0.166 | 0.002* |
| Which of the following tool is most commonly used to establish the causality of an Adverse Drug Reaction (ADR)? | 0.177 | 0.001* |
| Which of the following is the “WHO online databases” for reporting Adverse Drug Reactions (ADRs)? | 0.176 | 0.001* |
| ***Knowledge with Years of Experience*** | | |
| Which of the following defines an Adverse Drug Reaction (ADR) correctly? | 0.134 | 0.013* |
| Seventy-year-old man is taking Amiodarone for cardiac arrythmia and he developed Heart block as a side effect. Which of the following matches the type of adverse drug reaction in this patient? | 0.209 | < 0.001* |
| Fifteen-year-old boy was given injection of Benzylpenicillin for rheumatic heart disease prophylaxis and developed anaphylaxis as a side effect. Which of the following matches the type of adverse drug reaction in this patient? | 0.118 | 0.029 |
| The international center for Adverse Drug Reaction (ADR) monitoring is located in: | 0.278 | < 0.001* |
| Which of the following is the “WHO online databases” for reporting Adverse Drug Reactions (ADRs)? | 0.169 | 0.002* |
| ***Knowledge with Age*** | | |
| What is the best definition of Pharmacovigilance (PV)? | 0.134 | 0.013* |
| The international center for Adverse Drug Reaction (ADR) monitoring is located in: | 0.146 | 0.007* |
| **Attitude** | | |
| ***Attitude with Qualifications*** | | |
| I believe that ADR reporting should be made mandatory for practicing pharmacists. | 0.162 | 0.003* |
| It is important to report ADRs in order to answer the questions that may arise in my practice. | 0.135 | 0.012* |
| ***Attitude with Years of Experience*** | | |
| I am willing to spend enough time to discuss patient adverse drug reaction (ADR) on regular basis with my manager | 0.146 | 0.007* |
| There should be an incentive for pharmacists who are reporting ADR | 0.183 | < 0.001* |
| **Practice** | | |
| ***Practice with Qualifications*** | | |
| Have you observed any Adverse Drug Reactions in your practice in the past one year? | -0.165 | 0.002* |
| Have you ever reported any Adverse Drug Reactions in the past one year? | -0.176 | 0.001* |
| If yes, then to whom have you reported? | -0.176 | 0.001* |
| How many Adverse Drug Reactions cases have you reported to date in the past year? | 0.178 | < 0.001* |
| Did you take any training in Adverse Drug Reactions reporting at your work place? | -0.110 | 0.043 |
| Does your workplace encourage you to report an Adverse Drug Reaction? | -0.130 | 0.016 |
| Is Adverse Drug Reaction reporting mandatory at your current work place? | -0.119 | 0.027* |
| ***Practice with Years of Experience*** | | |
| Have you observed any Adverse Drug Reactions in your practice in the past one year? | -0.198 | < 0.001* |
| Have you ever reported any Adverse Drug Reactions in the past one year? | -0.295 | < 0.001* |
| If yes, then to whom have you reported? | -0.260 | < 0.001* |
| How many Adverse Drug Reactions cases have you reported to date in the past year? | 0.339 | < 0.001* |
| Does your workplace provide information regarding the procedure of reporting Adverse Drug Reactions? | -0.198 | < 0.001* |
| Did you take any training in Adverse Drug Reactions reporting at your work place? | -0.160 | 0.003* |
| Does your workplace encourage you to report an Adverse Drug Reaction? | -0.111 | 0.041* |
| Is Adverse Drug Reaction reporting mandatory at your current work place? | -0.224 | < 0.001* |
| ***Practice with Age*** | | |
| Have you ever reported any Adverse Drug Reactions in the past one year? | -0.155 | 0.004* |
| If yes, then to whom have you reported? | -0.181 | < 0.001* |
| How many Adverse Drug Reactions cases have you reported to date in the past year? | 0.174 | 0.001* |
| Is Adverse Drug Reaction reporting mandatory at your current work place? | -0.119 | 0.028* |
| **Barriers** | | |
| ***Barriers with Qualifications*** | | |
| All serious ADRs are already detected before registration of drug | -0.174 | 0.007* |
| Pharmacovigilance topic not included in pharmacy curriculum | -0.163 | 0.003* |
| ***Barriers with Years of Experience*** | | |
| All serious ADRs are already detected before registration of drug | -0.108 | 0.047* |
| **Suggestions** | | |
| ***Suggestions with Qualifications*** | | |
| There should be incentives for the pharmacist who perform the reporting | 0.145 | 0.007* |
| Availability of ADR reporting center in each hospital will enhance PV activity | 0.139 | 0.01* |
| Encourage all health professionals to report | 0.146 | 0.007* |
| Ease of access to ADR forms | 0.148 | 0.006* |
| Using information technology in facilitating ADR reporting in the country | 0.149 | 0.006* |
| PV should be taught in the pharmacy curriculum | 0.170 | 0.002* |
| ***Suggestions with Years of Experience*** | | |
| There should be incentives for the pharmacist who perform the reporting | 0.174 | 0.001* |
| ***Suggestions with Age*** | | |
| Direct ADR reporting by patients to national PV center | -0.130 | 0.016 |

* Significant (P < 0.05)

Bold values indicate statistical significance
